# Natural Antimicrobial Peptides as Inspiration for Design of a New Generation Antifungal Compounds

**DOI:** 10.3390/jof3030046

**Published:** 2017-08-26

**Authors:** Małgorzata Bondaryk, Monika Staniszewska, Paulina Zielińska, Zofia Urbańczyk-Lipkowska

**Affiliations:** 1 National Institute of Public Health-National Institute of Hygiene, Chocimska 24, 00-791 Warsaw, Poland; mbondaryk@pzh.gov.pl; 2 Institute of Organic Chemistry, Polish Academy of Sciences, 01-224 Warsaw, Poland; paulina.zielinska@gmail.com

**Keywords:** antifungal peptides, fungal pathogens, *Candida* species

## Abstract

Invasive fungal infections are associated with high mortality rates, despite appropriate antifungal therapy. Limited therapeutic options, resistance development and the high mortality of invasive fungal infections brought about more concern triggering the search for new compounds capable of interfering with fungal viability and virulence. In this context, peptides gained attention as promising candidates for the antimycotics development. Variety of structural and functional characteristics identified for various natural antifungal peptides makes them excellent starting points for design novel drug candidates. Current review provides a brief overview of natural and synthetic antifungal peptides.

## 1. Introduction

The increased prevalence of fungal infections is a consequence of the advances in medicinal technologies and therapies applied in critically ill patients [1]. Expanding immunocompromised population due to multi-organ failure or critical illness along with other clinical factors (Table 1) can increase the incidence of invasive fungal infections (IFI) [2,3,4,5,6]. IFI are of great concern due to high morbidity and mortality rates [7]. Over 90% of IFI-related deaths result from infections due to *Candida* spp., *Aspergillus* spp., *Cryptococcus* spp. and *Pneumocystis* spp. [7,8].

Currently available antifungal agents, based on their mode of action, can be divided to five classes: polyenes, azoles, echinocandins, pyrimidine analogs and allylamines [14,15,16,17,18,19]. Of these, allylamines are used against superficial infections, while the four remaining drug categories are effective against invasive mycoses [19]. Only two classes of systemic antifungal agents are considered to be non-toxic: triazoles (fluconazole and voriconazole) and echinocandins (caspofungin and micafungin) [14]. Therefore, current guidelines of European Society for Clinical Microbiology and Infectious Diseases (ESCMID) strongly recommend the use of fluconazole empirical therapy against invasive candidiasis in patients who recently underwent abdominal surgery and had recurrent gastrointestinal perforations [20]. Moreover, ESCMID strongly recommends the use of echinocandins for the targeted initial treatment of candidaemia [20,21].

Widespread use of antifungal agents in therapy and prolonged treatment often leads to the resistance development [19]. Resistance mechanisms were described for all commercially available antifungal agents (Table 2). Rising antifungal resistance is a major problem especially in case of fluconazole, a drug of choice for candidiasis treatment in AIDS patients [22]. Recent surveillance studies from various medical centers worldwide have documented the rise in echinocandins’ resistance; especially among *C. glabrata* isolates [14,19,23]. The need for novel, safe and effective antifungal agents increases in parallel with the expanding number of resistant fungal isolates [23,24]. Recent reports [25,26] have demonstrated the success of the combination of antifungal therapy regimens that utilize new generation antifungals (third generation azoles and lipopeptides—echinocandins) against severe mycoses. On the other hand, caspofungin and presumably other echinocandins have not been found to be an effective treatment for endemic mycoses, such as *Histoplasma capsulatum* [27,28], remaining one of the most feared complications of immunosuppression due to its significant morbidity and mortality [29,30]. In this regard, amphotericin B (AmB) remains highly active in vitro against the fungi responsible for *Histoplasma* infections [31,32,33]. Itraconazole is the primary azole for most endemic mycoses with its serum drug level maintained at the steady state of ≥ 0.5 mg/mL, which is particularly important in patients with severe infections [31,32,34,35]. Alternatively to itaconazole’s limitations (lack of tolerance, absorption problems or gastrointestinal intolerability) other azoles (fluconazole, voriconazole or posaconazole) are recommended [31,32]. As regards this, while the third-generation azoles have excellent in vitro and in vivo activity against *Histoplasma* [36,37], *H. capsulatum* develops resistance to fluconazole during therapy, leading to relapse [38,39]. Thus, *H. capsulatum*’s antigen concentrations in serum and urine should be monitored every three to six months to provide evidence that the maintenance therapy continues to suppress the progression of infection [38,39].

Standard antifungal therapies such as the azole family and AmB are not effective against *Pneumocystis* pneumonia (PCP), due to *P. jiroveci* (previously *P. carinii* f. sp. *hominis*), possibly due to the lack of ergosterol biosynthesis by these fungi [40]. While some reports suggest efficacy against PCP with the use of anidulafungin, caspofungin, or micafungin alone or in combination with a standard anti-PCP agent: trimethoprim-sulfamethoxazole or atovaquoneone alone [41,42], others did not report such effects [43,44,45,46]. As much as anidulafungin and caspofungin are more effective than micafungin in reducing cyst counts, they have other restrictions as described below. Findings of Cushion and Collins [47] suggest that the biofilm formation in the mammalian lung is a potential mechanism by which these fungi might resist the therapeutic interventions. Abrogation of echinocandins’ activity with the addition of human serum has been reported for *P. jiroveci* as well as *C. glabrata* [48]. Cushion and Collins [47] showed that *P. jiroveci* is more susceptible to the echinocandins in the suspension assay than in the biofilm systems.

Novel antifungal agent should have a broad-spectrum activity, target specificity, low toxicity, diverse mode of action, and no antagonistic effects with commercially available drugs [49,50]. Although such drug may be unattainable in reality, these properties should be used as guidelines in drug discovery [51]. Recently, there is an increased interest into peptides as a promising approach in discovery and development of novel antifungal agents. This review provides a brief overview of natural and synthetic antifungal peptides.

## 2. Antifungal Peptides

Development of novel antifungal compounds may overcome the problem of growing fungal resistance. In this context, peptides have promising properties, such as moderate immunogenicity as described below, strong antimicrobial activity, high specificity and affinity for targets, distinct mechanisms of action, good organ and tissue penetration and broad-spectrum activity [49,50]. Antifungal peptides have diverse action mechanisms, which include: (1) inhibition of DNA, RNA and protein synthesis; (2) binding to DNA or RNA; (3) membrane permeabilization; (4) inhibition of the cell wall synthesis and enzyme activity; (5) induction of apoptosis; and (6) repression of protein folding and metabolic turnover [49,53,54,55]. Antifungal peptides can be classified according to their mode of action and origin [56]. Based on their action mechanism, antifungal peptides can be divided into: (1) membrane traversing peptides that can cause pore formation or act on specific target such as β-glucan or chitin synthesis; and (2) non membrane traversing peptides that interact with the cell membrane and cause cell lysis [56]. According to their origin, peptides can be classified as natural compounds and synthetic molecules isolated from genetic or recombinant libraries or discovered from chemical libraries [56,57].

### 2.1. Natural Peptides

In higher organisms, antimicrobial peptides are part of the first line of defense against pathogens, while in microorganisms they are used in competition for nutrient resources [58,59]. Natural antimicrobial peptides usually show no or little toxicity against human cells and are stable in various conditions [60]. They generally possess common features, such as small size, overall positive charge, cationic and amphipathic nature [50,59]. Antimicrobial peptides can be arranged into different groups based on their length, sequence and structure [50]. The first group is formed by α-helical peptides, which mostly exhibit a random structure before interacting with the cell membrane, and well-defined structure afterwards [61]. Another large group consists of peptides that contain cysteine residues, form disulfide bonds and stable β-structures (sheet, hairpin, and barrel) [50,61]. Many other antimicrobial peptides (i.e., defensins) have mixed structure consisting of a cysteine-stabilized αβ-motif (CSαβ) with α-helix and a triple-stranded antiparallel β-sheet stabilized by four disulfide bonds [62].

The number of identified antimicrobial peptides exceeds 2700 and increases. Of these, over 900 peptides isolated from microbes, plants and animals display antifungal activity [50,63]. Natural antifungal peptides can be derivated from different sources, such as bacteria, archea, protists, fungi, plants and animals (Table 3).

As reviewed by Basak et al. [64], antimicrobial peptides display various levels of toxicity against mammalian cells. Interestingly, the ability of peptides to react specifically with the functional binding site of a complementary antibody known as antigenicity has been widely described [65]. The cytotoxic activity is related to the structural features of the peptide consisting of a cationic N-terminal sequence predicted to assume an amphipathic α-helical conformation (residues 1–18) and a C-terminal hydrophobic tail (residues 19–27). The hydrophobic tail is responsible for the peptide activity, since its analog, BMAP28 (1–18), which comprises the 18 N-terminal residues, showed a reduction in the neurotoxin 1-methyl-4-phenyl-1,2,3,6-tetrahydropyridine (MPTP) effect. Furthermore, BMAP28’s cytotoxicity requires an active metabolism of the target cells [66]. The cathelicidin-like peptide (SMAP-29) derived from myeloid sheep was strongly hemolytic against human erythrocytes [26,67]. Human cathelicidin (LL-37) was found to regulate the inflammatory response and chemo-attract the cells of the adaptive immune system into wound or infection sites, helping to neutralize the microorganism and promoting re-epithelization and wound closure [68,69]. Rondonin derived from the spider *Acanthoscurria rondoniae* with the antifungal activity showed no deleterious activities against human erythrocytes or Gram-positive and Gram-negative bacteria. Temporins identified in the skin secretion of the European red frog *Rana temporaria* [70], did not lyse human erythrocytes [71,72,73,74,75]. Bovine cathelicidins (BMAP-28) with the fungicidal activity was toxic for the mammalian tumor cells, inducing their apoptosis, and it also induced mitochondrial permeability, forming transition pores (MPTP), resulting in the release of cytochrome c.

Conventional antimicrobial agents (penicillin and beta-lactams), which are small organic molecules, are usually not immunogenic, although the extended longer treatment periods in immune compromised or critically ill patients can trigger an immune response [76]. Peptide (NZ2114), a40-residue long variant of plectasin, induced immune responses [8]. Contrariwise, smaller peptide, a pediocin AcH (class IIa bacteriocin) isolated form of *Pediococcus acidilactici*, displayed weak immunogenicity [14,15]. Cyclopeptide colistin (1155 Da) active against multiresistant *Acinetobacter baumannii* and administered despite its neuro- and nephrotoxic effects did not show immunogenic effects [17]. Moreover, insect derived proline-rich intermediate-size (i.e., 15–25 residues) antimicrobial linear peptides (PrAMPs) were usually weak immunogens. It was shown that they have to be cross-linked, oligomerized or coupled to larger carrier proteins or polymers to trigger a strong immune response [18,19].

Antimicrobial peptides (i.e., cathelicidins and β-defensins) either directly defend the pathogens [77] or stimulate inflammatory responses [78,79], leading to pro-inflammatory cytokine signaling [80] and toll-like receptor (TLR) activation [81]. This could end up in activating and recruiting immune cells to the site of infection and thus boosting the host immune defense. Many originally isolated antifungal peptides possess immunosuppressive activity blocking the recipient’s immune system, thus preventing a transplanted organ rejection [82,83]. They are also employed to slow down the immune response in patients suffering from certain immune disorders [84].

#### 2.1.1. Bacterial and Fungal Peptides

Antifungal peptides produced by microorganisms have distinct mechanism of action. Bacteria and fungi produce antifungal peptides that play a key role in competing for certain ecological niches [58]. For example, *Burkholderia cepacia* produces glycopeptides-cepacidines, which are active against a wide range of medically important yeast and filamentous fungi [86,136]. However, the presence of serum (50%) caused a decrease of their activity [136]. Syringomycins produced by *Pseudomonas syringae* pv. *syringae* or iturins produced by *Bacillus subtilis* are lytic peptides active against broad spectrum of yeasts and filamentous fungi [84,85,86,137]. Iturins affect membrane surface and create tension, which cause pore formation, leakage of K^+^ and other ions, resulting in cell death [56]. Despite iturins are highly active against *C. albicans*, *A. niger*, *Trichosporon* spp., and *Fusarium oxysporum*, their utilization in antifungal therapy is restricted because of hemotoxicity [86,89].

Antifungal peptides can also target vital processes, such as inhibition of 1,3-β-glucan or chitin synthesis. Inhibition of 1,3-β-glucan destabilizes cell wall, increases susceptibility to osmotic stress, and leads to cell death [107]. *Aspergillus aculeatus* produces aculeacins related to echinocandin family, which inhibit 1,3-β-glucan synthase [86]. Aculeacins are generally effective against *Candida* spp., except *C. tropicalis*. Contrariwise, they are not active against filamentous fungi [107]. In cells of *C. albicans*, aculeacins block morphogenetic transformation at concentrations below minimal inhibitory concentration (MIC) values [93]. These peptides were not clinically approved due to reduced antifungal activity and greater cytotoxicity compared to echinocandins [107]. Nowadays, targeting chitin is an appealing therapeutic challenge and its inhibitors are of potential interest for antimycotics development [107]. Chitin is an essential cell wall component of fungi, absent in vertebrates, that maintains the structural integrity of the fungal cell. Nikkomycins produced by *Streptomyces* species, acts as competitive analogs of chitin synthase substrate UDP-*N*-acetylglucosamine in pathogenic dimorphic fungi, particularly *Coccidioides* spp., *Histoplasma capsulatum* and *Blastomyces dermatidis* [91,138]. While nikkomycin Z exerts modest to poor activity against yeast and filamentous fungi, together with caspofungin or voriconazole, implements promising synergistic activity against *C. albicans* or both synergistic and additive activity against *C. neoformans* [86,90,138]. Interestingly, Li and Rinaldi [90] demonstrated that the growth of *C. albicans* and *C. parapsilosis* is dramatically limited under nikkomycin Z, but *C. tropicalis*, *C. krusei*, *C. glabrata* are resistant to this peptide. Unfortunately, the use of nikkomycins is limited due to unfavorable pharmacokinetics [107].

Another candidate in the development of new natural peptides addressing fungal virulence factors is pepstatin A. It was originally isolated from cultures of various species of *Actinomyces*. In fact, pepstatin A is a potent inhibitor of almost all types of aspartic proteases, and has been used to evaluate the virulence of the Sap family (Saps 1–10) [139,140,141,142]. As described latter, measurement of Sap7 proteolytic activity using the FRETS-25Ala library showed that Sap7 was a pepstatin A-insensitive protease [139]. Similar to Sap7, other proteins as follows: Sap9 and Sap10 are suggested to be less sensitive to pepstatin A [143]. The reason why *C. albicans* evolved Sap7 as well as Sap9, and Sap10 as pepstatin A-insensitive proteases remains to be clarified. It is possible that they are countermeasures against natural aspartic protease inhibitors. Pepstatin A inhibits pepsin, cathepsin D, renin, chymosin and HIV proteases [139,140,141,142]. Unfortunately, it cannot be used clinically, because of its metabolism in the liver and rapid clearance from blood [144,145].

#### 2.1.2. Plant Peptides 

Plants produce antimicrobial peptides as a part of defense mechanisms against environmental stress and pathogens. They primarily target pathogenic fungi; however, antibacterial and insecticidal activities are also reported [62]. Fungicidal mechanisms of most of these peptides remain to be fully elucidated [97]. Plant antimicrobial proteins contain a variety of peptides, including: defensins, thionins, lipid transfer proteins, hevein-like peptides, and others [62,97].

Plant defensins are small, highly stable, cysteine-rich peptides with antifungal properties [146]. They are active against *Fusarium* spp., *Saccharomyces cerevisiae* and *C. albicans* [86,146]. Plant defensins do not interact directly with phospholipids of plasma membrane and have distinct modes of action [146]. For instance, RsAFP2 produced by radish (*Raphanis stativus*) can induce fungal membrane permeabilization either by interacting with membrane’s glucosylceramides or by induction of reactive oxygen species (ROS) in *C. albicans* [86,146,147]. Furthermore, radish defensins RsAFP1 and RsAFP2 act synergistically with caspofungin against biofilm of *C. albicans* [62,94].

Thionins possess a conserved cysteine-rich domain with antimicrobial properties [54]. They act via membrane permeabilization and are active against pathogenic fungi, including *Fusarium* spp. and *Candida* spp. [97,98]. Aside from acting on fungal membrane, thionin-like peptide (*Ca*Thi) from *Capsicum annuum* fruits induces oxidative stress in *C. tropicalis*, which suggest a possible nuclear target for this protein [98].

Thaumatin-like (TL) proteins are another group of antifungal peptides that share significant amino acid homology to thaumatin. They can be isolated from corn, soybeans, rice, tomato, pumpkin and many other plants [148]. TL proteins display broad spectrum of activity and have distinct mechanism of action. For instance, osmotin, a TL protein from tabacco, is active against *Bipolaris* spp., *Fusarium* spp., *Phytophthora* spp. and *Trichoderma* spp. [99]. This protein can inhibit spore germination, cause perturbations in the regulation of fungal cell wall assembly or induce spore lysis [99,148]. Zeamatin, TL protein produced by corn seeds (*Zea mays*), is active against *C. albicans* and appears to act via rapid cell lysis [86,100]. This antifungal peptide acts synergically with nikkomycin Z and with azole drugs [100,148].

#### 2.1.3. Insect Peptides 

Due to the lack of an adaptive immunity, insects express a large number of antimicrobial peptides, which play a decisive role in the struggle against fungal pathogens [149]. Insect peptides are produced in fat body and secreted to hemolymph in response to fungal infection [50,149]. Cecropins are most renowned antimicrobial peptides isolated from insects [149]. They are a family of cationic antimicrobial peptides, first isolated from silk moth (*Hyalophora cecropia*). Cecropins exert their antifungal activity by targeting and lysing fungal plasma membrane [56]. They are highly fungicidal against blastoconidial cells of *C. albicans*, *Aspergillus* spp. and *Fusarium* spp. [86,149,150]. Stomoxyn is a cecropin constitutively expressed in gut epithelium of a stable fly (*Stomoxys calcitrans*). It displays structural similarities with cecropin A from *Hyalophora cecropia*, indicating similar mode of action on microbial membranes [54,151]. Stomoxyn has high activity against *Fusarium*, *Necteria*, *Trichoderma* and *Cryptococcus* species, however it exerts moderate to low activity against *Candida* spp. and *A. fumigatus* [101].

Melittin is another example of antifungal active compound produced by insects. It is a cationic, amphipathic hexacosa peptide found in the venom of the honeybee (*Apis mellifera*) [49,102]. Melittin exerts its antimicrobial activity either by inducing pore-formation in the plasma membrane or apoptotic actions [103]. It induces apoptosis in *C. albicans* through a ROS-mediated mitochondria- and caspase-dependent pathway [102]. Despite promising properties, melittin displays high hemolytic activity, which limits its clinical use [54].

Insect defensins are a class of small cationic cysteine-rich peptides active against bacteria and fungi [151]. Many of them, such as drosomycin, tenecin 3, holotricin 3 or termicin may belong to the insect defensin family [151]. Of these, drosomycin is a cysteine-rich peptide isolated from the hemolymph of fruit fly (*Drosophila melanogaster*), that selectively destroys spores and hyphae of filamentous fungi by partial lysis [104,152]. Drosomycin is a strictly antifungal peptide that acts in dose-dependent manner: at low concentrations, it delays hyphae growth and abnormal morphology of treated fungi, whereas at high concentrations it fully inhibits hyphae growth and spore germination [104]. To date, three antifungal glycine-rich peptides were isolated from insects: holotricin 3, antifungal peptide (AFP) and tenecin 3 [71]. Holotricin 3 and AFP, purified from the hemolymph of the *Holotrichia diomphalia* and *Sarcophaga peregrina* larvae respectively, are active against *C. albicans* [86,153]. Out of insect glycine-rich peptides, only tenecin 3 was studied in detail. This peptide is produced by *Tenebrio molitor* larvae and is active against *C. albicans* [71,105]. Internalization of tenecin 3 into the cytoplasm of *C. albicans* cells is required for cell death, which indicates number of involved intracellular targets [105]. Termicin and spinigerin are antimicrobial peptides isolated from a fungus-growing termite (*Pseudacanthotermes spiniger*) [154]. Termicin is a cysteine-rich peptide constitutively present in the hemocyte granules [154]. Structure of termicin contains α-helical segment and two-stranded antiparallel β-sheet forming a cysteine-stabilized αβ-motif, typically found in insect antifungal defensins [155]. It is active against yeasts and filamentous fungi. Interestingly, *Fusarium* and *Nectria* species are highly susceptible to termicin, whereas no activity was observed against *A. fumigatus* and *C. glabrata* [154]. Spinigerin is a 5-residue cysteine-free peptide, constitutively expressed in the termite’s hemocytes. It is effective against *C. albicans* and filamentous fungi [54,154]. Spinigerin has structural similarities with magainin 2, which suggests similar mode of action [54].

#### 2.1.4. Amphibian Peptides

Amphibian skin and tissue secretions are important source of biologically active peptides with high antifungal potential [156]. For instance, magainins are a family of antimicrobial peptides isolated from the skin of the African clawed frog (*Xenopus laevis*) [53]. Magainin 2 affects the plasma membrane by the formation of the transmembrane pores or ion channels and the leakage of essential metabolites [56,157]. It is active against *Candida* spp. and *S. cerevisiae* [106,107]. However, buforins are approximately 10 times more potent against a broad range of bacteria and fungi, compared to magainin 2 [106]. They display strong antifungal activity against *C. albicans*, *C. neoformans* and *S. cerevisiae* without any significant hemolytic activity [106]. Buforin I is a 39-amino acid residue peptide isolated from the stomach tissue of the Asian toad (*Bufo bufo garagriozans*). Buforin II is a 21-amino acid peptide derived from buforin I [55,106]. Despite higher antimicrobial activity, buforin II displays much weaker membrane permeabilization activity. Buforin II is more efficiently translocated across lipid bilayers than megainin 2, which suggest that this peptide targets intracellular components [158]. After penetrating cell membranes, buforin II binds to DNA and RNA, which inhibits cellular functions and result in the rapid cell death [49,55].

Dermaseptins are a super-family of α-helical amphipathic peptides isolated from the skin of frogs of the *Phyllomedusa* genus [110,159]. They interact with plasma membrane phospholipids, leading to permeabilization of cell membrane [110]. Dermaseptins display strong antifungal activity against yeasts and filamentous fungi [86,110,111].

Similar to other peptides of amphibia, temporins exert their antifungal activity by permeabilizing the cell membrane [55]. They were first isolated from the skin of European red frog (*Rana temporaria*) [109]. Temporins are active against *C. albicans*, *C. guilliermondii*, *C. tropicalis* and *S. cerevisiae* [55,109]. They preserve their antimicrobial activity in serum and are nontoxic to mammalian cells, which is a promising feature for drug development [160].

#### 2.1.5. Avian and Mammalian Antifungal Peptides

Mammalian AMPs include defensins and cathelicidins, having direct lytic properties against bacteria, fungi and viruses. Vertebrates’ defensins are divided into α-defensins, β-defensins and θ-defensins based on their disulfide bonds position, their structures and sequences [117]. Of these, α-defensins are presented in human, rabbit, rat guinea, pig and mouse. β-Defensins are present in human, bovine, pig, ovine, chicken, turkey, ostrich and king penguin [113,161,162]. α- and β-defensins are predominantly β-sheet structures stabilized by three disulfide bonds [86]. Defensins exert antimicrobial activity by forming pores in microbial membrane, which results in increased membrane permeability and the leakage of essential minerals and metabolites. Non-specific membrane disruption enables them to kill microbes without inducing resistance-gaining mutations [86,117,161].

α-Defensins are small arginine-rich peptides displaying antifungal activity. To date, six α-defensins HNP-1 to HNP-6 were identified in humans. Defensins from HNP-1 to HNP-4 are stored mainly in polymorphonuclear leukocytes and can be isolated from blood. HNP-5 and HNP-6 are expressed in Paneth cells of human small intestine [107,163]. HNP-1 and HNP-2 are active against *C. albicans* and *C. neoformans* [86,118]. However, their antifungal activity is rather low in comparison to α-defensins isolated from other species. For instance, rabbit defensin NP-1 is ten times more potent against *C. albicans* compared to HNP-1 [118]. Rabbit defensins (NP-1, NP-2 and NP-3) possess a broad spectrum of antifungal activity. They are active against *C. albicans*, *C. neoformans*, *A. fumigatus*, *C. immitis*, *Rhizopus oryzae* [117]. β-Defensins are expressed in epithelial tissues as an important element of the mucosal innate immune response against pathogenic fungi [120]. Human β-defensins (hBD) are a family containing at least six members active against *C. albicans* [164]. Bovine β-defensins, such as tracheal antimicrobial peptide (TAP) and lingual antimicrobial peptide (LAP), are active against *C. albicans* and *C. tropicalis*, respectively [118,119]. Avian β–defensins are potent antifungal agents directed against yeast and filamentous fungi. Gallinacins are cationic peptides isolated from chicken (*Gallus gallus*) [115]. They are usually 36-39 amino acids long and rich in lysine and arginine [86]. Gallinacins are generally effective against *C. albicans* and *S. cerevisiae* [112,113,114,115,116,117,118,119,120,121,122,123,124,125,126,127,128,129,130,131,132,133,134,135,136,137,138,139,140,141,142,143,144,145,146,147,148,149,150,151,152,153,154,155,156,157,158,159,160,161,162,163,164,165]. Additionally, chicken β-defensin-4 (sAvBD-4) and β-defensin-9 (sAvBD-9) display antifungal activity against *A. flavus* and *A. niger* [166]. Four β-defensins, named gallopavin-1 (GPV-1), THP-1, THP-2 and THP-3, were reported in turkey (*Meleagris gallopavo*) [167]. Of these, THP-1 and THP-3 are active against *C. albicans* [113]. Spheniscins, isolated from stomach content of king penguin (*Aptenodytes patagonicus*), are highly active against *C. tropicalis*, *Neurospora crassa* and *A. fumigatus* [114]. However, they exert low activity against *C. albicans* and are inactive against *C. glabrata* [114]. θ-Defensins are macrocyclic octadecapeptides found in Old World monkeys (i.e., *Rhesus monkey*) and orangutans [121]. Rhesus θ-defensin-1 (RTD-1) is a tridisulfide cyclic antimicrobial peptide derived from α-defensin-related precursors [122]. Defensins RTD-2 and RTD-3 are produced by homodimeric splicing of RTD1b and RTD1a and their in vitro activities are comparable with those of RTD-1. Defensins from RTD-1 to RTD-3 exert antifungal activity against *C. albicans* and *C. neoformans*, whereas RTD-4 and RTD5 display activity against *C. albicans*. Moreover, peptides from RTD-1 to RTD-3 and RTD-5 are at least threefold more potent than RTD-4 [121,122].

Mammalian cathelicidins represent a distinct class of cationic antimicrobial peptides with amphipathic properties [125]. Their antimicrobial potential is exerted by destabilization of microbial membranes [168]. Cathelicidins have highly conserved cathelin domain at the middle region of the pre-pro-peptide flanked by signal peptide at N-terminus [68]. Carboxy-terminal cationic antimicrobial domain is cleaved to produce a peptide with antimicrobial activity [116]. To date, LL-37 represents the only known human cathelicidin. It is produced by epithelial cells, macrophages, lymphocytes and neutrophils [168]. LL-37 displays antimicrobial properties and its expression increases in skin tissues infected with fungi [123]. It is active against *C. albicans*, *Malassezia furfur*, *Trichophyton rubrum* and *T. mentagrophytes* [123,168]. LL-37 exerts candidacidal activity by destabilization of plasma membrane and efflux of ATP and proteins. Moreover, by interacting with the cell wall components, LL-37 inhibits *C. albicans* adhesion [168]. Unfortunately, the clinical application of LL-37 has several limitations. Its biological activity significantly decreases in saliva or urine and its candidacidal potential is fully compromised in blood plasma [169]. Indolicidin is a tryptophan-rich bovine cathelicidin purified from the cytoplasmic granules of neutrophils [125]. Indolicidin disrupts the structure of cell membranes, via direct interaction with the lipid bilayers, in a salt-dependent and energy-independent manner [126]. It is active against different species of pathogenic fungi, including *C. albicans*, *C. neoformans*, *S. cerevisiae*, *Rhodotorula rubra* and *T. beigelii* [124,126]. Tritrpticin, a member of the cathelicidin family, is derived from a precursor protein in the porcine bone marrow and has high homology to indolicidin [127]. It is a small peptide of 13 amino acids comprised of a high concentration of tryptophan residues which are responsible for antimicrobial activity [56]. Tritrpticin has a weak inhibitory effect against *A. fumigatus* and *C. albicans* [127]. Protegrins are cysteine-rich β-sheet cathelicidins found in porcine leukocytes [128]. They are responsible for the formation of non-specific and poorly regulated channels in the cells of microorganisms [56]. To date, five members of protegrin family were identified (PG-1 to PG-5) [125]. Protegrins from PG-1 to PG-5 are effective against yeast-phase of *C. albicans* [128]. Moreover, PG-1 is active against *Candida* spp., *Pichia* spp., *R. rubra*, *S. cerevisiae* and *Penicillium* spp. [124]. Several avian cathelicidins were identified in chicken [170]. They share similar structures with their mammalian counterparts, each comprising of four exons, encoding a prepropiece consisting of a signal peptide, the cathelin-like domain (propiece) and the mature peptide [170]. Chickens produce at least four cathelicidins with antimicrobial activity: CATHL-1, CATHL-2, CATHL-3 and CATHB-1 [165,170]. Of these, chicken cathelicidin-2 (CATHL-2) displays highly antifungal properties against *C. albicans* causing a 1000-fold reduction in viable cells at 1.25 µM, and is fungicidal at 2.5 µM [116].

Histatins are another group of antimicrobial peptides found in the saliva of humans and some higher primates [129,171]. Histatin family consists of twelve structurally related members, of which histatin-5 (Hst 5) is the most potent antifungal peptide [129,171]. Hst 5 binds to the fungal cell wall proteins and glucans and it is taken up by cell via polyamine transporters. Inside the fungal cell, Hst 5 may affect mitochondrial functions, cause oxidative and osmotic stress [130]. Ion imbalance caused by osmotic stress leads to cell death [130]. It is active against *Candida* spp., *S. cerevisiae*, *C. neoformans* and *A. fumigatus* [129,131].

Lactoferrin is an iron-binding glycoprotein of the transferrin family [133]. It is a potent antimicrobial peptide found in neutrophil granules and in biological fluids, including milk, saliva or tears [134]. Lactoferrin is active against *C. albicans*, *C. glabrata*, *C. krusei* and *T. mentagrophytes* [134,135,172]. Moreover, it synergistically enhances the antifungal activity of azole drugs against *Candida* spp. [173]. Lactoferricin is a lactoferrin-derivated antimicrobial peptide with a broad spectrum of activity, including antimicrobial, antitumor, antiviral and immunological properties [133]. The two most important types of lactoferricins are human-derived Lfcin H and bovine-derived Lfcin B [56,133]. Lactoferricins exert antifungal potential by interacting with fungal plasma membrane and improving host defense [133]. They are active against yeasts, dermatophytes and moulds, such as: *Candida* spp., *Cryptococcus* spp., *Trichosporon* spp., *S. cerevisiae*, *Trichophyton* spp., *Penicillium* spp. and *Aspergillus* spp. [132,133].

Despite their great potential, active antifungal compounds can be extracted from natural sources in small amount that is not sufficient to carry out microbiological tests and makes the process unprofitable [174]. However, many natural antifungal peptides represent excellent starting points for future therapeutic agents. Therefore, synthesis or modification of existing natural antifungal peptides is a promising approach in drug development [53].

### 2.2. Interactions of Natural Antifungal Peptides with Other Antifungal Drugs

In most cases, natural antimicrobial peptides are secreted, and execute their antimycotic activity locally. However, mycoses in immunocompromised patients or neonates can lead to general infective shock (sepsis) and require a systemic therapy. Another challenging therapeutic issue is a progressive resistance of microbes residing in health care units. In both cases, peptides should be administered systemically in higher doses, seriously challenging homeostasis of the organism. There have been several successful approaches recently published that significantly overcome these drawbacks. One of the possible solutions is the use of antifungal peptides as components in the combination therapy with known antifungal drugs. In general, the experiment’s synergy designs a mixture of an antimicrobial peptide at sub-MIC concentration and an antifungal drug in several concentrations. The advantages are as follows: (1) reduction of antimycotic dose (unwanted side effects reduction); (2) prevention of drug resistance development; and (3) bioavailability and mechanistic polyfunctionality improvement. In this respect, the human antimicrobial peptides such as Lactoferrin (LF) and human cathelicidin LL-37 or Histatin 5 (His 5), with well documented broad antifungal activity are often selected for this type of studies. Among several possible, the antifungal azoles with low molecular weight and AmB, a potent and universal compound that is widely used against fungal infections in clinics, applied both topically (skin, nails, oral cavity) as well as in the cases of internal infections (urinary track or respiratory system), are in the center of interest. Although treatment with AmB is often recommended, its significant nephrotoxicity and infusion-related allergic reactions limits its application or even necessitates discontinuation of the treatment. 

Pioneering work of Wakabayashi et al. gave evidence that LF in form of intact protein and also shorter antimicrobial peptides obtained by LF enzymatic cleavage synergically interact with clotrimazole [175]. The minimum inhibitory concentration (MIC) of all azole antifungal agents tested was reduced by 1/4–1/16 in the presence of a sub-MIC level of each of these studied LF-hydrolizates. The authors were also the first to study interactions of LF and LF-related peptides with azole drugs, and to discover therapeutic potential of such mixtures on the azole-resistant *Candida* spp. [176] Interestingly, the addition of LF or LFcin B (cleavage product) at a sub-MIC resulted in a substantial decrease in the MICs of fluconazole and itraconazole against two highly azole-resistant strains. In particular, the MIC of fluconazole in the presence of LF for one of them (TIMM3317) was reduced 10^3^-fold (from > 256 to 0.25 µg/mL), without affecting the susceptible strains’ MICs. However, AmB and fluoropyrimidine did not show such a combined effect with the LF-related substances against the used collection of the azole-resistant *Candida* strains. Kuipers et al. studied the fungistatic effects of LF in combination with various antifungal drugs (fluconazole, AmB, and 5-fluorocytosine) against the clinical isolates of *Candida* [177]. The pronounced cooperative effect against the growth of *Candida* was observed for all three antifungal drugs, with LF-fluconazole combination being the most successful against azole-resistant *C. glabrata* isolate Y110. Recently Lai et al. studied interactions between iron chelators and antifungal drug AmB in treating infections induced by *Cryptococcus* [178]. In an extensive experiment involving antifungal drugs, potential iron chelators (azoles, e.g., fluconazole, itraconazole, voriconazole, etc.), antimicrobial peptide lactoferrin (LF), as well as compounds known as universal chelators (EDTA, deferoxamine mesylate, etc.) were used as adjuvants to AmB. It was found that all chelators except for deferoxamine mesylate increased the efficacy of AmB, reducing its dose, defined as MIC, two-fold. Four-fold dose reduction was detected for the combination of AmB with LF. Moreover, authors suggested that significant synergy between AmB and LF therapies aimed at disrupting iron homeostasis may have another advantage reducing incidence of anemia, a common side effect of AmB treatment [179].

Recently, new natural antifungal lipopeptide bacillomycin D (NH_2_-STNYNPE-OH) was isolated from *B. subtilis* B38 strain (soil) and revealed high potency in the tests against *Candida* species [180]. Subsequently, it has been used as an adjuvant in synergy studies with AmB. The highest synergistic effect in the anti-candidal activity of bacillomycin D combined with AmB was found to be for concentrations 32-fold lower than the MIC of bacillomycin D solely and only 4-fold lower than the MIC of AmB solely. Nevertheless, this mixture of a peptide and AmB displayed a limited hemo- and cytotoxicity at much higher concentration than that expressed by bacillomycin D. Evidently such strategy minimized peptide toxicity towards normal mammalian cells while preserving antifungal activity. This suggests also non-equal contribution of the two components into the synergistic process.

Another approach presented Mora-Avarro et al. who designed and synthesized a β-peptide (ACHC-β3hV-β3hK), potent against *Candida* spp., and evaluated the synergy with commonly used the azole-based antifungal drugs ketoconazole and fluconazole [181]. The combination of β-peptide mixed with either Fluc or Keto at sub-MIC concentration, expressed an enhanced biological effect against the fungal pathogenic model *C. albicans*, with the Keto/(β-peptide) being more effective than Fluc/(β-peptide) mixture. Although no synergistic behavior was found during the dual treatment of *C. albicans* biofilms, Fluc/(β-peptide) mixed with a constant β-peptide concentration of 32 μg/mL inhibited the biofilm for all Fluc concentration tested, whereas Fluc by itself was unable to inhibit the biofilm viability.

As mentioned before, the studies on the mechanism of action expressed by natural antimicrobial peptides revealed new molecular pathways that can be a rational tool in new drugs’ design. On the other hand, the production of the mitochondrial ATP is essential for the survival of the human parasitic protozoa *Leishmania*. Detailed studies on the mechanism of antimalarial activity conducted by Luque-Ortega et al, showed that His 5 in a relatively low dose (LC_50_ = 7.3 or 14.4 µM), acts as an inhibitor of mitochondrial ATP synthesis in two different life cycles, resulting in the cellular energy supply system’s collapse [182]. 

Authors suggested that, besides of being the first prospective leishmanicidal peptide with a defined nonstereospecific intracellular accumulation, His 5 might be also developed as a carrier of antimalarian or other drugs for the combined therapy. In continuation of this line, Park et al. recently proposed the His 5 modified nanoparicles as a new targeted delivery system for AmB [183]. Their structure contains water-soluble chitosan-conjugated to His 5 via pH-sensitive and AmB with a redox-sensitive linker. Both antifungal compounds were additionally PEG-ylated. In vitro and in vivo studies confirmed that besides complex structure and some problems with internalization, co-delivery of two antifungal compounds with different mode of action yielded strong synergistic effect against antibiotic susceptible and resistant *Candida* strains.

Summing up, although high synergy between two so structurally different compounds as peptides and heteroaromatic molecules was detected the mechanism of synergy is mostly unknown. Moreover, the final antifungal effect does not seem to be a simple sum of the effects of two molecules working simultaneously according to two different mechanistic pathways, and highly depends on the used compounds as well as the *Candida* species tested. Nevertheless, the idea of the application of natural antimicrobial peptides and their synthetic analogs as broadly active antifungal adjuvants seems to have a high potential in the development of new therapeutic tools.

### 2.3. Natural Antifungal Peptides in New Delivery Systems

General problems with the systemic application of small or medium size peptides created needs for designing the delivery systems that will improve safety, bioavailability, blood stability, and finally will allow a reasonable control of the release rate. In the recently published review on the delivery systems for the antimicrobial peptides, the majority of research was done for the peptides targeting Gram-positive or Gram-negative bacteria [184]. Systems for the delivery of antifungal peptides are scarce. On the other hand, such properties of antimicrobial peptides as good water solubility, high affinity to negatively charged microbial membranes, self-assembly at membrane interfaces and formation of amphipathic structures are advantageous for designing of novel delivery systems. In particular, these formulations might explore well-suited for human therapy the antimicrobial peptides such as lactoferricin, human cathelicidin LL-37, etc. For topical application, various hydrogels based on relatively inert inorganic and natural polymeric materials containing non-peptidic antifungals were designed [185]. An attempt was made to prepare the hydrogel containing the antimicrobial peptide LL-37 in the combination with chlorhexidine incorporated into the mesoporous silica (for structure see Table 2) [186]. Both antifungals were released gradually, reaching maximum release after about 200 h. The release rate could be slowed down by the incorporation of SH groups in the pore walls. Kong et al. reported the development of bioadhesive hydroxypropyl methylcellulose hydrogel containing Histatine 5 for topical treatment of opportunistic oral candidiasis [187]. The in vivo test of a mouse model of oral candidiasis revealed very good biocompatibility, lack of toxicity, as well as anti-inflammatory and wound-healing properties of Hst-5 in such formulation.

Summing up, biocompatible hydrogels or non-toxic nanoparticles are interesting matrices for the confinement of natural antifungal peptides. Obviously, there is a niche for nanoformulations of antifungal peptides as new generation nanomedicines for topical applications.

### 2.4. Synthetic Peptides and Peptide Derivatives

Recent research [54] has focused on the application of various methods yielding new molecular entities, with enhanced antimicrobial activity, reduced toxicity and lower costs of production. There are several approaches for design of the synthetic peptides including: (1) template-based design; (2) rigorous biophysical modeling; and (3) virtual screening [2,188]. Template-based strategy is known as de novo design of peptide analogs based on a template peptide with known antifungal activity [2]. Biophysical modeling examines peptide structures in hydrophobic environments or by modeling peptides at the atomic level. Virtual screening studies can be used when synthesis and antifungal tests are expensive [188]. Template-based strategy (de novo design) is often most convenient approach in peptide design [2]. Sequence modifications of natural antifungal peptides aim to enhance their activity and reduce toxicity. Activity screening is usually investigated by examining peptide variants that were designed based on the template [2,188]. For example, three libraries of Sap inhibitors were designed and synthesized by modifying the structure of microbial pepstatin A at the P3, P2 and P2’ positions [189]. Based on the knowledge of the ability of Sap2 to cleave peptide substrates bearing an Arg side chain at the P2’ position, in the first library, the hydrophobic Ala at the latter position was replaced by Lys, Orn, Dab, or Arg, that are cationic at physiological conditions. These inhibitors showed selectivity against Sap3. Similarly, the second library includes analogs in which the Val in P2 position was replaced by positively charged Lys or hydrophobic Nle, Leu or Abu. These inhibitors explored the specificity of the Sap S2 pocket and were active against Sap5–6. The third library was synthesized based on the knowledge of the enlarged S3 pocket of Sap isoenzymes. In five analogs Val at the P3 position was replaced by Leu, Phe, p-MePhe, Tyr, or O-MeTyr. All these variants showed the same potency against Sap3 [189]. Recently, two de novo designed antifungal peptides, VS2 and VS3, have been described [190]. These peptides were active against *Candida* spp., *C. neoformans*, *A. niger*, *F. oxysporum* and *N. crassa*. Peptides caused a direct membrane permeabilization and induced necrosis by causing an intracellular accumulation of reactive oxygen species [190].

Tryptophan-rich peptides such as indolicidin and tritrpticin are potent antifungal agents [124,191]. Tryptophan residue has a strong preference for the interfacial region of the lipid bilayers of the yeast cell membrane [191]. Therefore, the number of tryptophan residues along with amphipathic structure may enhance antifungal activity of peptides [2]. Inspired by these properties, many research groups designed various tryptophan-rich synthetic peptides with improved antifungal activity. For instance, a substitution of hydrophobic amino acids in PMAP-23, antimicrobial peptide isolated from porcine, with tryptophan enhanced anticandidal potential of created analogs [192]. Similar findings were reported with the activity of KU2, a hybrid peptide designed by selecting and fusing peptide fragments from KABT-AMP and uperin templates [2]. Increased numbers of tryptophan residues incorporated in the KU2 sequence improve its antifungal properties by enhancing the anchoring of peptide to the fungal membrane [2]. 

Ideally, therapeutic peptide antifungals should retain their activity in physiological conditions; however, efficiencies of many peptides are greatly reduced or diminished under high salt concentrations [193]. To overcome this problem, some studies were focused on the design of salt-resistant antifungal peptides. For instance, halocidin-derived di-K19Hc is active against yeast and filamentous fungi, such as *C. albicans*, *C. neoformans*, *A. niger*, *A. terreus* and *F. oxysporum* [194]. Di-K19Hc is a promising structure of drug development as it retains antifungal potential in elevated salt concentration, however structural motifs required for salt-resistance remain to be studied [194]. One described strategy to increase salt resistance of antimicrobial peptides is to replace histidine or tryptophan residues with the bulky amino acids β-naphthylalanine and β-(4,4′-biphenyl)alanine [193]. However, substitution with β-naphthylalanine residues enhances membranolytic properties of peptides and their hemolytic activity [193].

Conventional antifungal agents target viability, exerting fungistatic or fungicidal effect. This matter possesses a high degree of selective pressure which leads to the emergence of resistant strains [19,172]. Therefore targeting virulence traits is an attractive alternative approach for the development of new antifungals [172,195,196]. Targeting fungal virulence factors has several advantages including: (1) preservation of the host microbiome; (2) expansion of the number of potential drug targets; (3) development of new antifungals with novel modes of action; and (4) and weaker selective pressure [195,196]. Recently, antifungal activity of synthetic peptide KSL-W has been described [197]. KSL-W reduces growth of *C. albicans* and attenuates its virulence in terms of reduced adherence and growth on engineered human oral mucosa. The peptide also modulates proinflammatory cytokine secretion and downregulates gene expression of Toll-like receptors and β-defensisns in human oral mucosa [197]. Among fungal virulence traits, targeting of candidal secreted aspartic proteinases (Saps) seems to be a particularly promising approach in drug development [172].

Ability to form biofilm represents another promising virulence factor of *C. albicans* [172]. Biofilm formation modulates antifungal resistance of *Candida* cells to conventional antifungal agents, which may carry serious clinical consequences. Biofilm resistance can be up to 1000-fold higher compared to that of planktonic cells [198]. Therefore, new antifungal peptides should be active against cells within biofilms [2,195]. One class of such synthetic molecules is designated as kaxins [199]. The most potent peptide of this group, named dF21-10K, at concentration of 10x MIC was able to completely eradicate preformed biofilm of *C. albicans* and *C. tropicalis* [199]. Recent report on antibiofilm activity of uperin analogs indicated that enhanced antibiofilm activity correlates with the increased cationicity of peptides [2]. According to the latter study, numbers of positive charged residue enhance the interaction of peptides with the fungal cell wall [2].

Although it sounds promising to target fungal virulence traits, several disadvantages can be expected from this drug discovery approach. One drawback of this strategy is that *C. albicans* express different virulence factors during infection and inhibiting one of them may be not enough to clear the infection [200]. As virulence factors are pathogen-specific, anti-virulence drugs will display a narrow spectrum of action being effective against a single pathogen. Therefore, pathogen-specific anti-virulence agents will require the rapid and accurate diagnosis of fungal infections prior to their use [195,196].

### 2.5. Dendrimeric Peptide Mimics

**Figure 1:**
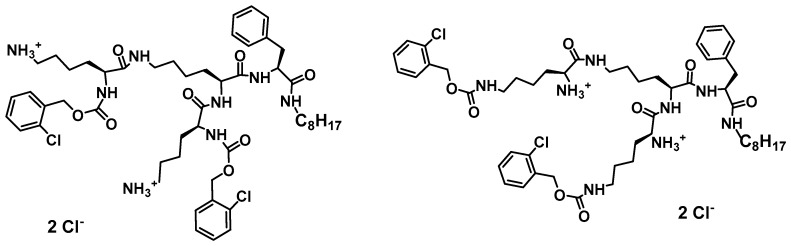
Examples of diastereomeric structures of branched lipopeptides providing different distribution of cationic and lipophilic centers [214].

Considering the conclusions in the above section, several groups adopted another approach to search for new innovative drugs; that is, based on design of molecules containing multiple ligands. Such compounds could engage multiple receptors or provide high local ligand concentration in physiological environment, both features beneficial for the fungi eradication process. Molecules that have proven their usefulness in such strategy are dendrimers. Dendrimers (tree-like molecules) are molecules with radially distributed layers of branches called generations (Figure 1) [201]. These polymer-like nanomolecules are synthesized by well-controlled synthetic methods and therefore are characterized by a high monodispersity as well as precisely defined number of terminal chemical groups that can be further modified. They can serve as templates for covalent or noncovalent assembling of multiple ligands and are also known for expressing so called “dendrimeric effect”, i.e., non-linear enhancement of properties, e.g., drug potency or bioavailability [202,203,204]. The most studied are the polyamidoamine dendrimers terminated with amine groups (PAMAM-NH2) developed by Tomalia et al. [205]. Under physiological conditions, they are cationic, water-soluble and permeate easily through various cellular membranes. Due to the presence of numerous amide bonds, they are regarded as macromolecular peptides or protein mimics. Dendrimers of higher generations have globular shape and contain intramolecular hydrophobic cavities suitable for inclusion of small drug molecules. Therefore, they are well suited as carriers of covalently attached antifungal drugs or for preparation of physical mixtures with drug molecules that are poorly soluble in body fluids due to their hydrophobic structure. Another group of dendrimers is constituted by polylysine dendrons (PLL), first proposed by Tam et al. [206], as so called Multiple Antigenic Peptides (MAPs) [206,207,208,209,210]. They were used for the preparation of carrier-allocated antigens for development of vaccines technology [211,212]. PLL dendrons have been used as scaffolds for presenting multiple peptide epitopes to the immune system. Such molecules consist of an inner matrix constructed from several layers of lysines connected via α- and ε-amino groups [213]. Two amino groups of lysines that are located at the surface of molecule can be orthogonally substituted by one or two different ligands (e.g., peptide chains). Among several advantages of such construction is the enhanced enzymatic stability.

#### 2.5.1. Dendrimers as Carriers of Bioactive Substances and Solubility Enhancers

In the area of antifungal research, PLL dendrons have recently been used as scaffolds carrying two or four copies of 10-residue peptide (RGRKVVRRKK) that exhibited a potent activity against *P. aeruginosa*, and much lower activity against fungi [215]. Comparison with monomer and retrodimer showed that both divalent and tetravalent dendrons expressed the non-linear enhancement of activity (dendritic effect) against three reference *Candida* strains and two clinical isolates of *C. albicans*. The tetravalent dendron named B4010 displayed better antifungal properties than the reference antimycotic drugs—natamycin and amphotericin B. Surprisingly, this highly cationic compound is non-hemolytic and non-toxic to mice by intraperitoneal (up to 200 mg/kg) or intravenous (up to 100 mg/kg) routes of administration. Moreover, it retains activity under high salt conditions as well as in a presence of enzymes like trypsin, blood serum and tear fluids. It displays very fast carpet mechanism with membrane dissipation and ion channels opening, typical for numerous antimicrobial peptides that are characterized by a set of random conformations.

Systematic development by fungi resistance against available drugs on one side, and lack of new antifungal drugs prompted research towards searching for new molecular targets. In particular, new molecular entities expressing novel and/or multiple mechanisms of action seem to be an interesting alternative. Along this line, synthesis of four generations of poly (amidoamine) (PAMAM) dendrimers carrying multiple benzenesulfonamide moieties was accomplished (G0–G4). These dendrimers were studied as prospective inhibitors of carbonic anhydrases present in pathogenic bacteria, as well as in fungi or protozoa. Although no direct correlation between dendrimer generations (i.e., molecular size and number of sulfamoyl moieties) and inhibitory potency was found, fungal carbonic anhydrase of *C. glabrata* qualified as β-class-Can2—was selectively inhibited only by G0 dendrimer (incorporating 4 sulfamoyl moieties) with a KI at the level 24.0 nM. Belonging to the same class, CgCA anhydrase was selectively inhibited only by G2 dendrimer (KI of 66.4 nM). Dendrimers of G1 and G3 generation were devoid of any inhibitory effect (KI > 342 nM) [216]. Transition metal cations (Cu(II) and Zn(II) dynamically complexed by multiple chelating sites located within properly functionalized dendrimer tree are subject of several recent studies. In particular, second generation polypropyleneimine dendrimers (PPI) terminated with various bis-1,8-naphthalimide derivatives have been found as a promising new lead compounds designed as antimicrobial therapy against some pathogenic Gram-positive and Gram-negative bacteria and the yeast *C. lipolytica* [217].

Very often, the inadequate aqueous solubility of some drugs is the major factor limiting their efficacy in medical therapy. However, in the presence of poly(amidoamine) (PAMAM) dendrimers, their bioavailability is often significantly improved. It is believed that observed enhancement of the drug solubility is due to the intermolecular interactions (intermolecular hydrogen bonding and hydrophobic interactions) between the intramolecular cavities of higher generation PAMAMs and included drug [218]. Recently, Winnicka et al. [219], have shown that lipophilic drug ketoconazole formulated as a solution mixture with the second generation amino (PAMAM-NH_2_) or hydroxyl (PAMAM-OH) groups terminated compounds exhibited 16-fold increase of anti-*Candida* potency in relation to the drug alone [219]. Moreover, antifungal activity of designed hydrogel with PAMAM-NH_2_ dendrimers measured by the plate diffusion method was definitely higher than that of pure ketoconazole hydrogel and that of commercially available product. Similarly, PAMAM-NH_2_ and PAMAM-OH dendrimers of generation 2 (G2) and 3 (G3) significantly influenced bioadhesive properties, viscosity and yield stress of polyacrylic acid polymer hydrogels with clotrimazole [220]. Structurally different polypropyleneimine dendrimers of the fourth generation, PPI-NH_2_ G4 (32 primary amino groups at the molecular surface), expressed innate broad antimicrobial and anti-*Candida* activity [221]. However, when reduction of the cytotoxicity typical for poly-cationic molecules was attempted by substitution of 4 or 32 amino groups by relatively inert maltose units, molecular activity was selectively shifted towards Gram-positive genera along with concomitant loss of activity against Gram-negative bacteria and *Candida* strains.

#### 2.5.2. Inherent Activity of Dendrimeric Peptide Mimics and Their Antifungal Formulations 

The so-called template-based strategy (de novo design) is often the most convenient approach in peptide design [2]. Sequence modifications of natural antifungal peptides aim to enhance their activity, reduce toxicity and decrease affinity to enzymatic digestion. While natural antimicrobial peptides were originally proposed to act via membranolytic mechanism, the antifungal mechanism for these peptides is generally more complex and often involves intracellular targets. According to evidence collected by van der Weerden et al. [71], very few peptides that demonstrate both antibacterial and antifungal activity act via the same mechanism. Therefore, the antifungal activities of peptides cannot be anticipated from studies on their antibacterial activities and have to be assessed separately. Vast majority of naturally occurring antimicrobial peptides share several characteristic structural features that define their biological profile. Usually, they are amphiphilic linear 10–50 amino acid sequence molecules with overall positive charge and significant number of amino acids with hydrophobic side chains. Such structure promotes nonspecific interactions on contact with negatively charged microbial cell membranes [61]. Destabilization of microbial membranes leading to loss of membrane potential, membrane thinning, channels or pores formation, leads to suppression of a vital cell functions and finally induces cell death. According to common knowledge, gained mostly from NMR studies, in order to maximize electrostatic interactions at membrane interphase, native conformation of linear peptides changes from random to α-helical or β-sheet (or other), what results in developing of an active structure consisting of positively charged and hydrophobic domains [222]. It appears that such structural features, i.e., cationic character and amphipathic structure might be efficiently modeled using three-dimensional structure of dendrimeric peptides. Indeed, such considerations prompted us to initiate development of so called “non-sequential pharmacophore” approach [223]. Essentially, low molecular weight branched peptides (1000–2500 a.u.) were synthesized that due to orthogonal substitution of basic amino acids—lysine, ornithine, arginine, β-aminoalanine, etc.—yielded molecules with variable distribution of cationic and lipophilic side chains (Phe, Ala, Val, etc.). Antimicrobial activity measured as minimal inhibitory concentration (MIC) in the range from 124 to low micromolar assayed against Gram-positive and Gram-negative bacteria and *Candida* reference strains confirmed that indeed, the active conformations mimicking that of natural antimicrobial peptides were generated [224,225]. Advantage of such methodology is that the positioning of residues critical for expressing antifungal activity, compound’s purity and monodispersity can be precisely controlled during peptide synthesis. Further development towards more hydrophobic molecules revealed that small dendrimeric lipopeptides acquired selective activity against *Candida* spp. and expressed low hemotoxicity (Figure 1) [214]. Moreover, this group of small, branched lipopeptides revealed multiple mode of action. The membranolytic mechanism, typical for majority of natural peptides was confirmed at sub-MIC and MIC concentration by direct observation of *Candida* cells morphology under scanning microscope. At higher dendrimer concentrations dendrimers expressed inhibitory activity against fungal β(1,3)-glucan synthase and ability to open potassium channels. Thus, not only fungal cell membrane but also cell wall was targeted by these lipodendrimers. 

**Figure 2:**
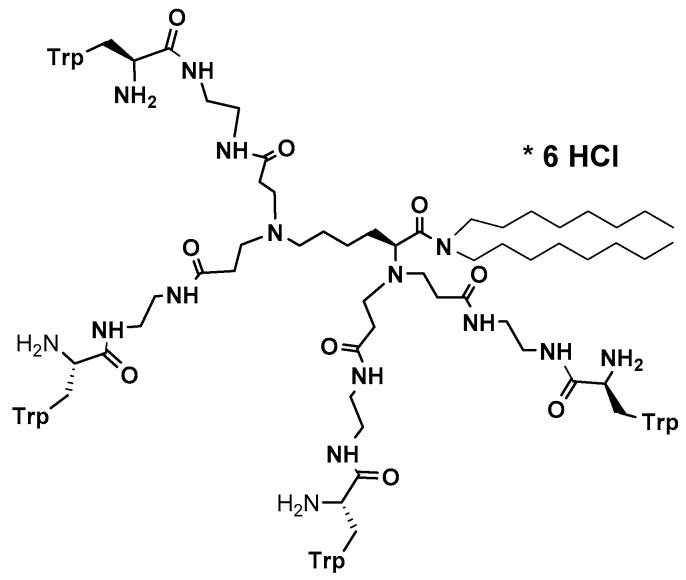
Rich in tryptophan residues dendrimeric peptide **D186** [227].

Multiple mechanism of action has been proved as valuable/indispensable quality of new drugs mostly because development of various resistance mechanisms by living cells is significantly hindered if drugs express multiple mode of action. With this respect, natural antimicrobial peptides can be of great inspiration. Recently, it has been found that the high content of tryptophan in peptides like lactoferricin B, indolicidin, tritrpticin, etc. is beneficial for their antimicrobial efficiency [191,226,227]. Along this line, dendrimer D186 carrying four Trp residues and dioctylamide group (Figure 2) as structural mimic of natural cationic amphiphilic peptides was designed and showed for the first time the ability to inhibit several aspartyl proteases (*SAP*s) that modulate virulence potential of *Candida* by influencing cell morphology in terms of adhesive ability and pathogenicity [227].

Consequently, this peptide prompted design of a group of eight new, structurally variable peptide dendrimers covered with several copies of bare or *N*-methylated tryptophanes and their subsequent evaluation confirmed multiple mode of action of this group of compounds as well [228].

**Figure 3:**
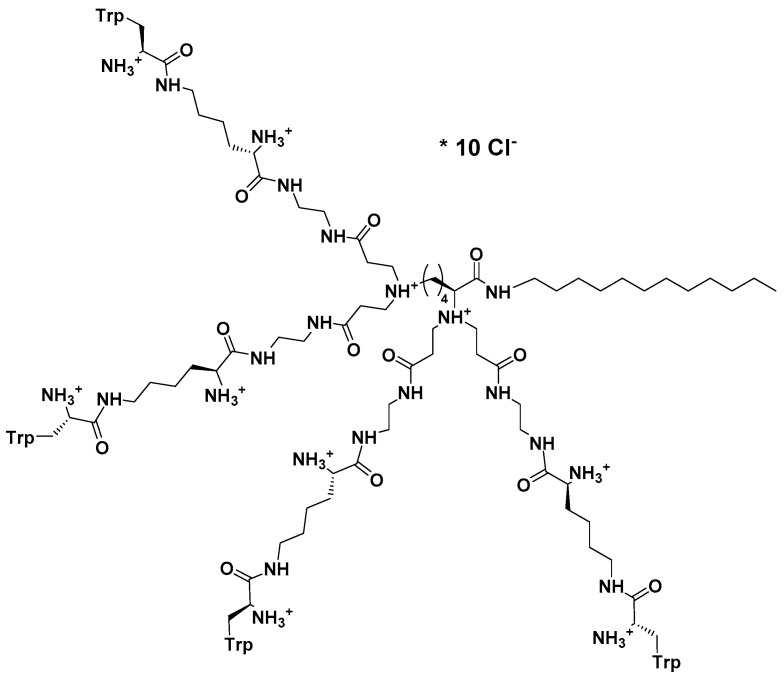
Structure of cationic Trp-rich dendrimer **14 [228]**.

Of these, most active dendrimeric peptide **14** (Figure 3) was effective against young and mature biofilm of *C. albicans*. This peptide inhibited biofilm formation by modulating cell polarization leading to the defects in hyphal formation and adhesion. Dendrimer’s activity against mature biofilm was exerted by acting negatively on the germination and reducing hyphae formation. In addition to membranolytic properties, dendrimer **14** at sub-MIC concentration (16 μg/mL) induced apoptosis and necrosis in higher concentrations. Thus, this group of Trp-rich branched peptide mimics interacts with components of the cell surface (cell wall and cell membrane) in multiple ways and therefore fits into the desired profile of modern multifunctional antifungals.

## 3. Conclusions

Emerging fungal resistance to conventional therapies necessitates the development of novel antifungal strategies. In this context, peptides gained attention as alternative potential antifungal agents. Peptides are highly effective, relatively safe and well tolerated [229]. Despite these features, only few antifungal peptides are used in antifungal therapy [56]. There are several problems limiting peptide use, such as hemolytic activity, low bioavailability, possible aggregation, instability, high cost of production, rapid turnover in the human body, poor ability to cross physiological barriers, and loss of activity in high salt concentrations [49,50,56,60]. As a result, therapeutic use of antifungal peptides is significantly limited. However, unfavorable properties limiting peptide use could be overcome by chemical optimization and new delivery strategies [56]. Moreover, the wide variety of structural and functional characteristics identified for various natural antimicrobial peptides makes them extremely promising source of ideas in design the novel antimycotic drug candidates. In particular, application of dendrimers as scaffolds for assembling well defined macromolecular polyvalent molecules or synthesis de novo of per se active linear and branched peptide mimics makes them extremely promising for use as new generation antifungals—in part because an ultimate result of these modifications were compounds that expressed multiple mode of action. As emphasized recently in several studies, for these peptides and peptide-mimics to be used, their modes of antifungal action must be well understood. Hopefully, all these efforts will result in the development of a novel class of antifungal agents to their full potential.

## Figures and Tables

**Table 1:** Risk factors for the development of invasive fungal infections (IFI).

Risk Factors	References
Abdominal surgery/recent major surgery	[3,5,9,10]
Deep burns	[11,12]
Diabetes mellitus	[9,10,12]
Dialysis	[12,13]
Disturbance of natural skin or mucosal barriers	[10,12]
Exposure to broad-spectrum antibiotics	[3,10,12]
Extremes of age	[5,10]
HIV/AIDS	[9,13]
Immune disorders	[5,11]
Local disorders of the gastrointestinal tract	[5,10,12]
Long-term catheterization	[3,5,13]
Malignancies	[3,5]
Mechanical ventilation	[10,13]
Parenteral nutrition	[5,10,13]
Premature very low birth weight infants	[11]
Prolonged hospitalization	[12]
Renal failure	[9,11,12]
Solid organ or bone marrow transplantation	[5]
Treatment with corticosteroids	[3,5]
Use of immunosuppressive drugs	[5,10,12]

**Table 2:** Five major groups of antifungal agents.

Group Name	Group Member/s	Mode of Action	Resistance Mechanism	References
**Fluorinated Pyrimidine Analogs**	Flucytosine (5-FC)	Inhibition of RNA and/or DNA synthesis	Deficiency in enzymes involved in pyrimidine salvage and 5-FC metabolism Mutations in *FCA1*, *FUR1*, *FCY21*, *FCY22*	[15,52]
**Polyenes**	Nystatin Natamycin Amphotericin B	Alteration of the membrane function by binding of ergosterol (depleting cells of ergosterol)	Defects in the *ERG3* gene (lowered ergosterol content in cell membrane) Altered membrane composition–substituted nonergosterol cytoplasmic membrane sterols and lipids (e.g., zymosterol, squalene) Capsule enlargement of *C. neoformans*	[18,52]
**Echinocandins**	Caspofungin Micafungin Anidulafungin	Alteration of cell wall biosynthesis by inhibition of β(1,3)-glucan synthase Fks1p or Fks2p	Mutation in the *FKS1* and *FKS2* genes Lack of 1,3-β-glucan in the cell wall of *C. neoformans*	[16,19]
**Allylamines**	Terbinafine Naftifine	Inhibition of the ergosterol biosynthesis by inhibition of squalene epoxidase (Erg1) and/or accumulation of toxic sterol intermediates	Modification of drug target (missense mutation or substitution in the *ERG1*) Degradation of the naphthalene ring contained in terbinafine	[16,19]
**Azoles**	Fluconazole Posoconazole Voriconazole	Inhibition of cytochrome P450 14α-lanosterol demethylase (encoded by *ERG11*) in ergosterol biosynthesis pathway	Mutations or overexpression of *ERG11* Reduced accumulation of the azoles inside fungal cell (reduced uptake of azoles, efflux via ABC transporters) Tolerance to methylated sterols via mutation in *ERG3*	[16,19,52]

**Table 3:** Natural peptides active against fungi ^1^.

Name	Origin	Sequence or Molecular Formula	Mode of Action	Reference
**Bacterial Peptides**				
Syringomycin	*Pseudomonas syringae* pv. *syringae*	MSLQANTAPVFADEQQTDAPTWPDRAADPSVRLSLLATGNSLPVVIEPTADGLDPVQWASARREAIETLLCRHGAVLFRGFDLPSVAAFEGFAEALSPGLHGTYGDLPKKEGGRNVYRSTPYPEREMILYHNESSHLESWPRKQWFFCEQPSRVGGATPLADIRQVLAYLPKEVVERFESKGLLYSRTFTAGVEPSWESFFGTSERSVIEQRCREQGTDFEWLDGDTLQLRTQCPAVITHPFTGERCFFNQVQLHHPYCMGEELREDLLDMFGPDRLPRLVSYGDGSAIEDPVMALIGEAYEACAVRFEWRKGDVVMLDNMLAAHARDPYEEPRLIVVAMGEMTARGDVWQPA	Cell lysis	[84,85,86,87,88]
SyrP protein				
Iturin A	*Bacillus subtilis*	KIYGVYMDRPLSAGEEVRMMAAVSAEKREKCRRFYHKEDAHRTLIGDMLIRTAAAKAYGLDPAGISFGVQEYGKPYIPALPDMHFNISHSGRWIVCAVDSKPIGIDIEKMKPGTIDIAKRFFSPTEYSDLQAKHPDQQTDYFYHLWSMKESFIKQAGKGLSLPLDSFSVRLKDDGHVSIEL	Cell lysis	[86,88,89]
Nikkomycin	*Streptomyces* spp.	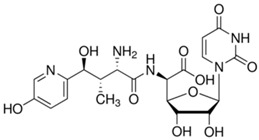	Inhibition of chitin biosynthesis	[90,91,92]
Pepstatin A	*Streptomyces* spp.	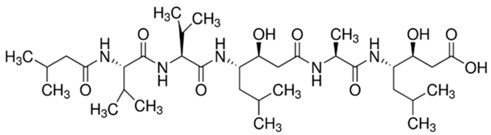	Inhibition of aspartic proteases	[92]
**Fungal Peptides**				
Aculeacin A	*Aspergillus aculeatus*	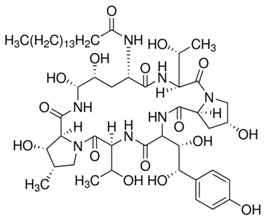	Inhibition of 1,3-β-d-glucan synthase	[92,93]
**Plant Peptides**				
**Defensins**				
Rs-AFP1	*Raphanus sativus*	QKLCERPSGTWSGVCGNNNACKNQCINLEKARHGSCNYVFPAHKCICYFPC	Membrane permeabilization	[62,88,94,95,96]
Rs-AFP2		QKLCQRPSGTWSGVCGNNNACKNQCIRLEKAWGSCNYVFPAHKCICYFPC		
**Thionins**				
*Ca*Thi	*Capsicum annuum*	KEICCKVPTTPFLCTNDPQCKTLCSKVNYEDGHCFDILSKCVCMNRCVQDAKTLAAELIEEEFLKQ	Membrane permeabilization	[88,97,98]
**Thaumatin-like (TL) proteins**				
Osmotin	*Nicotiana tabacum*	ATIEVRNNCPYTVWAASTPIGGGRRLDRGQTWVINAPRGTKMARVWGRTNCNFNAAGRGTCQTGDCGGVLQCTGWGKPPNTLAEYALDQFSGLDFWDISLVDGFNIPMTFAPTNPSGGKCHAIHCTANINGECPRELRVPGGCNNPCTTFGGQQYCCTQGPCGPTFFSKFFKQRCPDAYSYPQDDPTSTFTCPGGSTNYRVIFCPNGQAHPNFPLEMPGSDEVAK	Cell wall perturbations; spore lysis	[86,88,99,100]
Zeamatin	*Zea mays*	AAVFTVVNQCPFTVWAASVPVGGGRQLNRGESWRITAPAGTTAARIWARTGCKFDASGRGSCRTGDCGGVLQCTGYGRAPNTLAEYALKQFNNLDFFDISLIDGFNVPMSFLPDGGSGCSRGPRCAVDVNARCPAELRQDGVCNNACPVFKKDEYCCVGSAANDCHPTNYSRYFKGQCPDAYSYPKDDATSTFTCPAGTNYKVVFCP	Cell lysis	
**Insect Peptides**				
**Cecropins**				
Stomoxyn	*Stomoxys calcitrans*	RGFRKHFNKLVKKVKHTISETAHVAKDTAVIAGSGAAVVAAT	Cell lysis	[54,88,101]
Melittin	*Apis mellifera*	GIGAVLKVLTTGLPALISWIKRKRQQ-CONH_2_	Proapoptotic activity	[102,103]
**Defensins**				
Drosomycin	*Drosophila melanogaster*	DCLSGRYKGPCAVWDNETCRRVCKEEGRSSGHCSPSLKCWCEGC	Cell lysis	[86,88,104]
Tenecin 3	*Tenebrio molitor*	DHHDGHLGGHQTGHQGGQQGGHLGGQQGGHLGGHQGGQPGGHLGGHQGGIGGTGGQQHGQHGPGTGAGHQGGYKTHGH	Unknown	[71,88,105]
Holotricin 3	*Holotrichia diomphalia*	YGPGDGHGGGHGGGHGGGHGNGQGGGHGHGPGGGFGGGHGGGHGGGGRGGGGSGGGGSPGHGAGGGYPGGHGGGHHGGYQTHGY	Growth inhibition	
Termicin	*Pseudacanthotermes spiniger*	ACNFQSCWATCQAQHSIYFRRAFCDRSQCKCVFVRG		
**Amphibian Peptides**				
Magainin 2	*Xenopus laevis*	GIGKFLHSAKKFGKAFVGEIMNS	Disrubtion of plasma membrane	[106,107,108]
Buforin I	*Bufo bufo garagriozans*	AGRGKQGGKVRAKAKTRSSRAGLQFPVGRVHRLLRKGNY	Cell lysis	[55,106]
Buforin II		TRSSRAGLQFPVGRVHRLLRK		
Temporin A	*Rana temporaria*	FLPLIGRVLSGIL	Cell lysis	[56,88,109]
Dermaseptin-1	*Phyllomedusa hypochondrialis*	GLWSTIKNVGKEAAIAAGKAALGAL	Membrane permeabilization	[86,88,110,111]
**Avian Peptides**				
**Avian β-defensins**				
Gallinacins	*Gallus gallus*	GRKSDCFRKSGFCAFLKCPSLTLISGKCSRFYLCCKRIWG	Cell lysis	[86,88,112,113,114,115]
β-defensin-4		IVLLFVAVHGAVGFSRSPRYHMQCGYRGNFCTPGKCPHGNAYPGLCRPKYSCCRW		
THP-1	*Meleagris gallopavo*	GKREKCLRRNGFCAFLKCPTLSVISGTCSRFQVCC		
Spheniscin-1	*Aptenodytes patagonicus*	SFGLCRLRRGFCAHGRCRFPSIPIGRCSRFVQCCRRVW		
**Cathelicidins**				
Cathelicidin-2	*Gallus gallus*	LVQRGRFGRFLRKIRRFRPKVTITIQGSARFG	Cell lysis	[88,116]
**Mammalian Peptides**				
**α-defensins**				
HNP-1	*Homo sapiens*	ACYCRIPACIAGERRYGTCIYQGRLWAFCC	Cell lysis	[86,88,117,118]
HNP-2		CYCRIPACIAGERRYGTCIYQGRLWAFCC		
NP-1	*Rabbit bocaparvovirus*	MSSRHSPYPRKTSGDTTGSKTSWASSGSRENKGNHKNPSFSTASRPFLTRQQKKEILKPRALRKDPPKVFCATHRADSPDAPAVCGFFWHSNRIAGKGTDWIFTRGKQLFQERAKNNVIDWDMARDLLFSFKRECDQWYRNMLYHFRLGEPCDKCNYWDGAYRKYCARVNADYEKEINATSASQELTDEEAAAALDAAMADASH		
**β-defensins**				
HBD-1	*Homo sapiens*	DHYNCVSSGGQCLYSACPIFTKIQGTCYRGKAKCCK	Cell lysis	[88,118,119,120]
HBD-2		TCLKSGAICHPVFCPRRYKQIGTCGLPGTKCCKKP		
HBD-3		GIINTLQKYYCRVRGGRCAVLSCLPKEEQIGKCSTRGRKCCRRKK		
HBD-4		ELDRICGYGTARCRKKCRSQEYRIGRCPNTYACCLRK		
Tracheal antimicrobial peptide (TAP)	*Bos taurus*	MRLHHLLLALLFLVLSAWSGFTQGVGNPVSCVRNKGICVPIRCPGSMKQIGTCVGRAVKCCRKK		
Lingual antimicrobial peptide (LAP)		VRNSQSCRRNKGICVPIRCPGSMRQIGTCLGAQVKCCRRK		
**θ-defensins**				
RTD-1	*Macaca mulatta*	GFCRCLCRRGVCRCICTR	Cell lysis	[88,121,122]
**Cathelicidins**				
LL-37	*Homo sapiens*	LLGDFFRKSKEKIGKEFKRIVQRIKDFLRNLVPRTES	Destabilization of plasma membrane	[88,123]
Indolicidin	*Bos taurus*	ILPWKWPWWPWRR	Destabilization of plasma membrane	[88,124,125,126]
Tritrpticin	*Sus scrofa*	VRRFPWWWPFLRR	Destabilization of plasma membrane	[88,127]
Protegrin-1		RGGRLCYCRRRFCVCVGR	Destabilization of plasma membrane	[88,124,128]
**Histatins**				
Histatin-5	*Homo sapiens*	DSHAKRHHGYKRKFHEKHHSHRGY	Osmotic stress	[88,129,130,131]
**Lactoferrin-derived peptides**				
Lactoferricin H	*Homo sapiens*	GRRRSVQWCAVSQPEATKCFQWQRNMRKVRGPPVSCIKRDSPIQCIQA	Disruption of plasma membrane	[88,132,133,134,135]
Lactoferricin B	*Bos taurus*	FKCRRWQWRMKKLGAPSITCVRRAF		

^1^ Natural peptides showing activity against pathogenic fungi.
